# Damage prediction and improvement method based on cutting mode of circular empty hole

**DOI:** 10.1038/s41598-024-61599-x

**Published:** 2024-05-17

**Authors:** Huifeng Qin, Yan Zhao, Hailong Wang, Lijie Ge, Xiao Tong

**Affiliations:** 1 Hebei University of Architecture, Hebei, 075000 China; 2 School of Mechanics and Civil Engineering, China University of Mining and Technology (Beijing), Beijing, 100083 China; 3 Hebei Provincial Key Laboratory of Civil Engineering Diagnosis, Reconstruction and Disaster Resistance, Hebei, 075000 China; 4 Zhangjiakou BIM Engineering Technology Innovation Center, Zhangjiakou, 075000 China; 5 School of Earth Science and Engineering, Hebei University of Engineering, Handan, 056038 China

**Keywords:** Mode of injury, Holes space utilization rate, Dynamic evolution of stress, The proportion of rock breaking energy, Prediction and improvement, Climate-change ecology, Cardiology

## Abstract

Based on the theory of empty hole effect of cutting blasting, the Hopkinson effect and Saint–Venant principle are integrated to establish a two-dimensional calculation model of dynamic stress evolution of the holes wall, and then the dynamic fracture mechanism and damage distribution mode of the rock mass in the cutting area under the action of longitudinal waves are predicted. The results of the calculation and numerical simulation are verified by experiments, and the results show that: The time-varying stress function of the circular cavity wall conforms to the periodic dynamic evolution of the trigonometric function, and the theoretical calculation is consistent with the simulation results. Through the calculation of the round holes cut model and the square empty hole cut model, the change of the shape of the holes in the cut area changes the failure form of the surrounding rock mass. The circular empty hole wall is affected by the stress wave to produce "interval ring" destruction, and the effect of the reflected stretch wave is inhibited. The large range of rock mass in the square empty hole wall produces tensile and shear failure, and the rock mass collapses inward under the influence of the second stage stress. Among them, the empty space utilization rate of the square empty hole model is about 8.5 times that of the circular holes model. Vibration monitoring in the center of the cutting area shows that the vibration effect of the circular empty hole is larger than that of the square empty hole, and the proportion of rock breaking energy is lower.

## Introduction

The use of explosives is a key technology of tunnel excavation in mine rock roadways, but the accurate control of the damage to the rock mass is a difficult problem to solve. The interaction between blasts holes is complicated^1^. Accurate analysis of the mechanism of surrounding rock destruction in the face of the explosion is the key to controlling the damage of the groove. The traditional arrangement of cut blast holes has certain limitations. Due to the large vibration produced by fragments in the blast holes, the surrounding rock rupture in the central area of the cutting holes is not sufficient, and the explosive energy cannot be effectively used. An empty hole can provide a weak free surface and guide the expanding rock mass cracks, improving the rock mass fragmentation effect.

Based on the above problems, domestic and foreign scholars have studied the stress and damage distributions of explosion-influenced rock masses in empty hole groove areas under different parameters. For example, Chen et al.^2^ through theoretical calculation and numerical simulation, analyzed the fracture movement mechanism guided by the tangential stress concentration of the open holes under the synergistic action of dynamic and static loads. To accurately calculate the concentrated stress magnitude generated by the empty hole effect, Yang et al.^3^ studied the propagation trends of cracks at different inclination angles (including 45°) under different initial static stress fields. According to the specific parameters of an empty hole, Zhang et al.^4^ performed a theoretical analysis and calculation on the basis of the stress concentration effect, crushing space principle and free surface effect and verified the effectiveness of the blasting parameters combined with calculation examples. Regarding the crack damage evolution of an empty hole in the blasting groove area, Meng et al.^5^ established the rock mass damage tension constitutive model plate with LS-DYNA, studied the rock mass damage caused by the empty hole effect, and concluded that the existence of the empty hole significantly improved the tensile stress and stress concentration coefficient near the empty hole. In this work, the stress wave propagation during cut blasting was analyzed, and through dynamic blasting experiments, the bastings in specimens with circular empty hole, square empty hole and cut holes were analyzed and compared, and the propagation of the corresponding stress waves and explosion cracks were observed^6^. Sun et al.^7^ determined that the holes arrangement will affect the rock breaking effect of the groove area and determined the most suitable empty hole spacing.

According to the above research, the change in the holes spacing of a rock mass will cause stress concentration at the empty hole. Different empty hole shapes will change the concentration degree and path of stress, and a reasonable empty hole shape will effectively control the damage distribution of the rock mass in the groove area. In terms of the theoretical study of the evolution process of empty hole wall stress, the existing theoretical calculation model of stress around an empty hole cannot accurately reflect the stress evolution. In this study, the dynamic change in stress at the empty hole wall was established to study the influence of the dynamic mechanism of the tensile–shear stress at the empty hole section. Based on this, the layout method of a square empty hole was proposed, and more accurate results were obtained through this new method of calculation and simulation.

## Two-dimensional damage theory models

**Figure 1:**
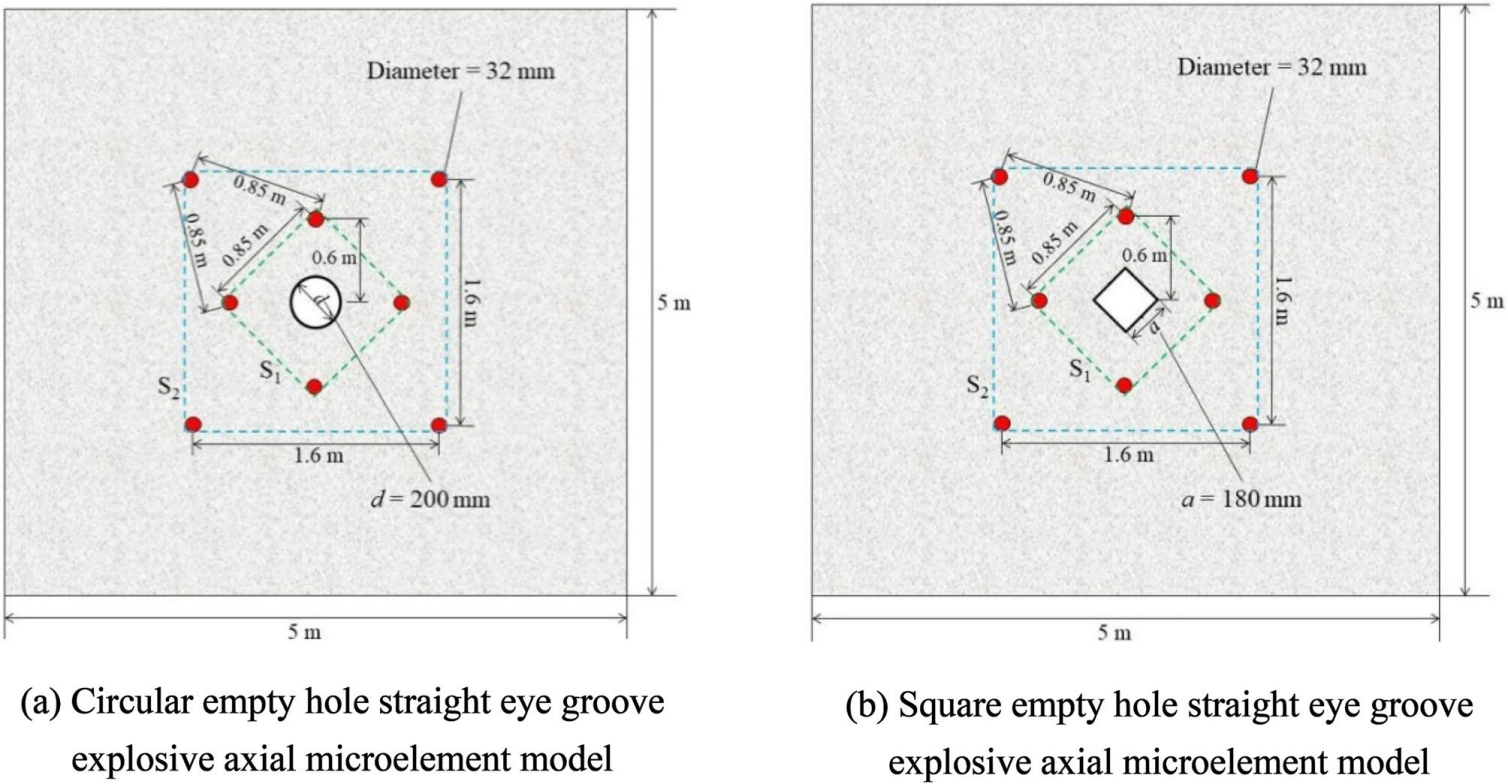
Layouts of empty hole in the cut section.

The two-dimensional model is a theoretical analysis and calculation model of the dynamic distribution of the energy of a rock mass under blasting load based on elastic mechanics theory^8^. This study refers to holes data, as shown in Table 1. Figure 1a,b ar based on the principle of expansion, the stress concentration effect, the free surface effect, and elastic and structural mechanics analysis^9^.

**Table 1:** Blast holes parameters in the cut section.

Number of holes	Breeching /(mm)	Empty hole/(mm)	*l*_1_/(m)	*l*_2_/(m)	*l*_3_/(m)	*l*_4_/(m)
8	32	200/180	0.6	0.85	0.85	1.60

Note: *l*_1_ is the spacing between 1 and 0 blast holes, *l*_2_ is the spacing between 1 and 2 blast holes, *l*_3_ is the spacing between 1 and 5 blast holes, and *l*_4_ is the spacing between 5 and 6 blast holes.

When the explosive explodes, the stress spreads in the form of a cylindrical wave. In the far area of blasting, the radius of curvature of the wave array surface is infinite, which can be used to determine the equivalent load of the stress wave generated by the explosion at an empty hole. The groove holes are divided into two sections: the four groove holes in S_1_ are the first section and the four groove holes in S_2_ are the second section. Since the theoretical calculation model is different from the actual engineering situation, the mechanical model is based on the following assumptions for convenient research:

1. The surrounding rock material is uniformly elastic‒plastic and isotropic;
2. The stress load generated by blasting is approximately equivalent to the uniform load applied on the empty hole wall;
3. The microelements are evenly distributed and have normal physical strengths;
4. The impact of local stress is not considered;
5. The cohesion and friction angle of the rock mass are not considered (These two coefficients are defined as independent quantities in the calculation of "void shape effect", and the critical state of rock mass material before failure is explored on the basis of elasticity. In this failure mode, internal friction Angle and cohesion can be introduced as other influencing factors in the calculation model).

After calculation, the parameters of the holes in the cut section are as follows:

### Foundation principle of groove blasting with empty hole

- Stress concentration effect: When the material is affected by an external force, the force will vary due to the no uniformity of the material^2,10^.
- Free surface effect: When the stress wave generated by the explosion of the explosive goes to the free surface of the empty hole, the stress wave causes tension and compression of the rock between the empty hole and the blast holes^11^.
- Broken-expansion theory: When the explosive explosion impacts the rock mass, due to the uncertainty of the blasting action, the rock mass produces several random transverse and longitudinal cracks. The expansion of the rock mass requires a reduction in the surrounding open spaces, and the expansion coefficient is generally expressed by Kp.

### Two-dimensional thin-walled ring model

**Figure 2:**
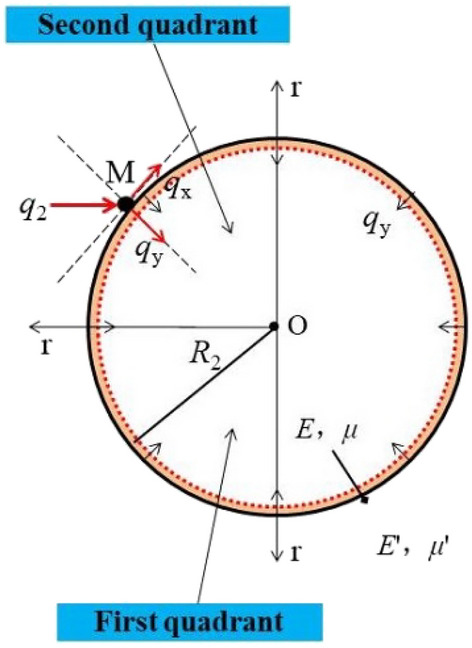
Force model of thin-walled ring.

The stress wave concentration at an empty hole wall under the action of a stress wave is a dynamic process, and the conventional calculation method for stress concentration around circular empty hole cannot accurately reflect the holes wall stress change process. When the holes wall reaches the critical damage value, it is subjected to the force characteristics of a circle^12,13^. A thin-walled ring model was established the as shown in Fig. 2^14^.

When the first segment of the stress wave attenuates in the rock mass, the attenuation coefficient *γ* (*γ* = 2 − *ν*_d_), and *q*_2_ is the equivalent uniform load at the empty hole wall (the empty hole curvature is much less than the curvature radius of the equivalent carrier surface, and the stress wave propagation speed is very fast). After the empty hole wall transmits the tensile wave and reflection wave, the model can be equivalent to a holes with one end fixed to determine the uniform load static equivalent system^6^. In the S_1_ area, the radius of the empty hole is *R*_2_, the blast holes distance from the empty hole is *L*_1_, and the thickness of the thin wall is ∆*R* (0 < ∆*R* ≤ *δ*, *δ* calculated from the deformation of the rock mass). Due to *L*_1_ ≥ ∆*R*, the stress concentration effect at the empty hole, the stresses on either side of the thin wall and the load on the ring are zero, in line with the Saint–Venant principle.

The relationship among the elastic modulus of the thin-walled ring in S_1_ *E*', the shear modulus *G*', and the lateral contraction coefficient *ν*' is* G*' = *E*'/2 (1 + *ν*'). For the elastic modulus of the thin-walled ring *E*, the shear modulus *G*, the lateral contraction coefficient *ν*, *ν* is related to *μ*_d_, and *μ*_d_ is the dynamic Poisson's ratio: *ν* = *μ*_d_/(1 − *μ*_d_). According to the relevant literature, *μ*_d_ = 0.8 *μ*, where *μ* is the dynamic Poisson's ratio.

The thin-walled ring as shown in Fig. 4. The equivalent load *q*_2_ produced by blasting acts on the thin wall in both quadrants, producing the radial stress *q*_y_ and the tangential stress *q*_x_ component on the thin wall of the second quadrant. According to the calculation, the expression of the stress component *q*_y_ is *q*_y_ = *q*_2_ sin *θ*, where *θ* ∈ (0, 0.5π) (*θ* is positive from r_1_ to r_2_).

From the Saint–Venant principle, at a distance relative to the thin wall, (*σ*'_*r*_)_*r*→*l*_ = 0 and (*σ*'_*θ*_)_*r*→*l*_ = 0 where *L*_1_ ≥ *l* ≥ *R*, C' = 0, and the displacement of the thin-walled ring should be the same as that of the mass beyond the ring, resulting in (*σ*_*r*_)_*r*_ = *R*2 + ∆_*R*_ = (*σ*'_*r*_)_*r*_ = *R*2 + ∆_*R*_, (*u*_*r*_)_*r*_ = *R*2 + ∆_*R*_ = (*u*'_*r*_)_*r*_ = *R*2 + ∆_*R*_, and [*A*/(*R*_2_ + ∆*R*)^2^] + 2 *C* = *A*'/(*R*_2_ + ∆*R*)^2^.

After the blasting load, the compaction of the compacted circular mass is greater than that of the outer ring mass due to the stress concentration effect of the circular empty hole, so *G* > *G*', *ν* < *ν*', and *E* > *E*', so *m*' < 1. After calculation, *m*' is 0.903^4^.

The stress components *σ*_*r*_ and *σ*_*θ*_ on the thin-walled ring are given as follows:

1 $$\left\{ \begin{gathered} \sigma_{r} = \gamma q_{2} sin\theta \frac{{[1 + m^{\prime}(1 - 2\nu_{{\text{d}}} )]\frac{{(R2 + \vartriangle R)^{2} }}{{r^{2} }} - (1 - m^{\prime})]}}{{[1 + m^{\prime}(1 - 2\nu_{{\text{d}}} )]\frac{{(R2 + \vartriangle R)^{2} }}{{R2^{2} }} - (1 - m^{\prime})]}} \hfill \\ \sigma_{\theta } = - \gamma q_{2} sin\theta \frac{{[1 + m^{\prime}(1 - 2\nu_{{\text{d}}} )]\frac{{(R2 + \vartriangle R)^{2} }}{{r^{2} }} + (1 - m^{\prime})]}}{{[1 + m^{\prime}(1 - 2\nu_{{\text{d}}} )]\frac{{(R2 + \vartriangle R)^{2} }}{{R2^{2} }} - (1 - m^{\prime})]}} \hfill \\ \end{gathered} \right.$$

### Two-dimensional theoretical model of square empty hole wall

**Figure 3:**
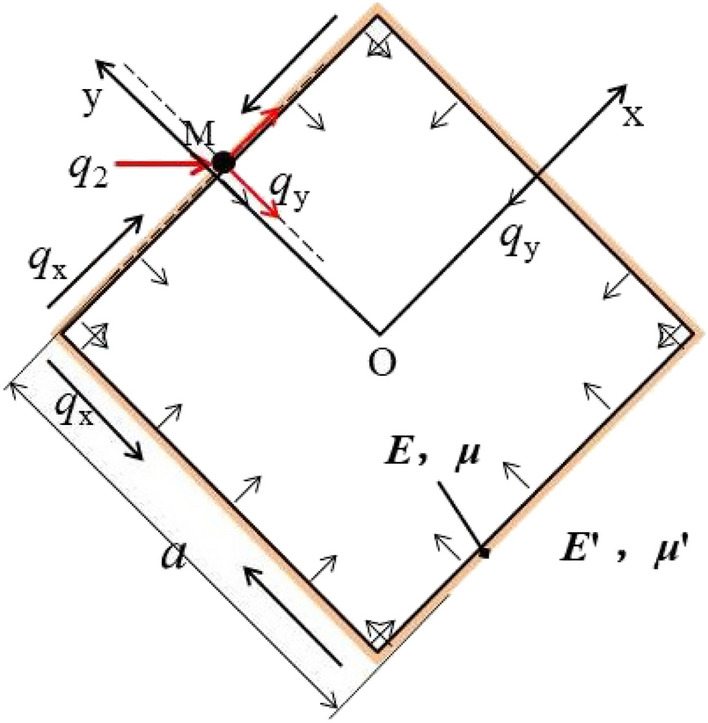
Force model of a square empty hole in an infinite domain.

To follow the theory of fragmentation and expansion, the empty hole of the improved model adopts a square empty hole, keeping the cross-sectional area of the empty hole consistent. According to the calculation, *a* = π^0.5^*R*_2_. To build the model shown in Fig. 3, the side length is *a*.

The model is defined as the problem of a compressed square empty hole in a plane. The model introduces the complex change function to accurately determine the stress around holes^15–20^. As shown in Fig. 3, the z-plane is perpendicular to plane XOY at the square empty hole wall. The square empty hole wall is subjected to the equivalent uniform load *q*_2_ from blasting. Assuming that the uniform load simultaneously acts on the square empty hole wall, the force decomposition of point M is shown. The square empty hole wall is affected by *q*_x_ parallel to the wall and *q*_y_ perpendicular to the wall, where *q*_x_ = 0.5π^0.5^ *q*_2_ and *q*_y_ = 0.5π^0.5^ *q*_2_. Because the square empty hole is subject to the uniform distribution load from all sides, *q*_x_ cancels out in the square empty hole rock mass, and then the two-dimensional plane system of the square empty hole wall becomes the pressure problem of only a uniform load distribution along* q*_y_.

Using a complex variable function solution, the conformal mapping function z is introduced:

2 $${\text{z}} = \omega \left( \zeta \right) = A\left( {\frac{1}{\zeta } + \alpha 1\zeta^{3} + \alpha 2 \zeta^{7} } \right)$$

Expression for calculation of square empty hole wall stress:

3 $$\begin{aligned} \sigma \theta & = 4{\text{Re}} \left[ {B + \frac{{3\frac{s1}{A} - 14B\alpha_{2} e^{4i\theta } }}{{ - e^{ - 4i\theta } + 3\alpha 1 + 7\alpha_{2} e^{4i\theta } }}} \right] \\ & = 4B + 4\frac{{\left( {\frac{{3s_{1} }}{A} - 14B\alpha_{2} \cos 4\theta } \right)\left[ {3\alpha_{1} + \left( {7\alpha_{2} - 1} \right)\cos 4\theta } \right] - 14B\alpha_{2} (1 + 7\alpha_{2} )\sin^{2} 4\theta }}{{\left[ {3\alpha_{1} + \left( {7\alpha_{2} - 1} \right)\cos 4\theta } \right]^{2} + (1 + 7\alpha_{2} )\sin^{2} 4\theta }} \\ \end{aligned}$$

The final expression of *σ*_*θ*_ is:

4 $$\sigma \theta = \gamma q_{2} (1 + \nu_{d} )\frac{{3n1n2 - 14n1\alpha 2\cos 4\theta - 14\alpha 2(1 + 7\alpha 2)\sin^{2} 4\theta }}{{n1^{2} + (1 + 7\alpha 2)\sin^{2} 4\theta }}$$

Since *θ* = 0.25 × *k* + 0.5π (where *k* = 1, 2, 3), *q* (*θ*) = *γ q*_2_. With *q* (*θ*) = *γ q*_2_ × *B* = 0.25 × *q* (*θ*) × (1 + *ν*_d_), B' − *i* × C' = 0.5 *q* (*θ*) × (1 + *ν*_d_) e^2*i θ*^, *n*_1_ = 3 *α*_1_ + (7 *α*_2_—1), and *n*_2_ = 3 *s*_1_/(*A* × *B*), the holes opening edge length ratio is known to be 1, so *α*_1_ = − 1/6 and *α*_2_ = 1/56. Assuming a value for *θ* (*θ* = 0, π/4, π/2), *A* = 1.392*a* and | *r* |= 0.025*a*^21^.

## Two-dimensional model theory and analysis

**Figure 4:**
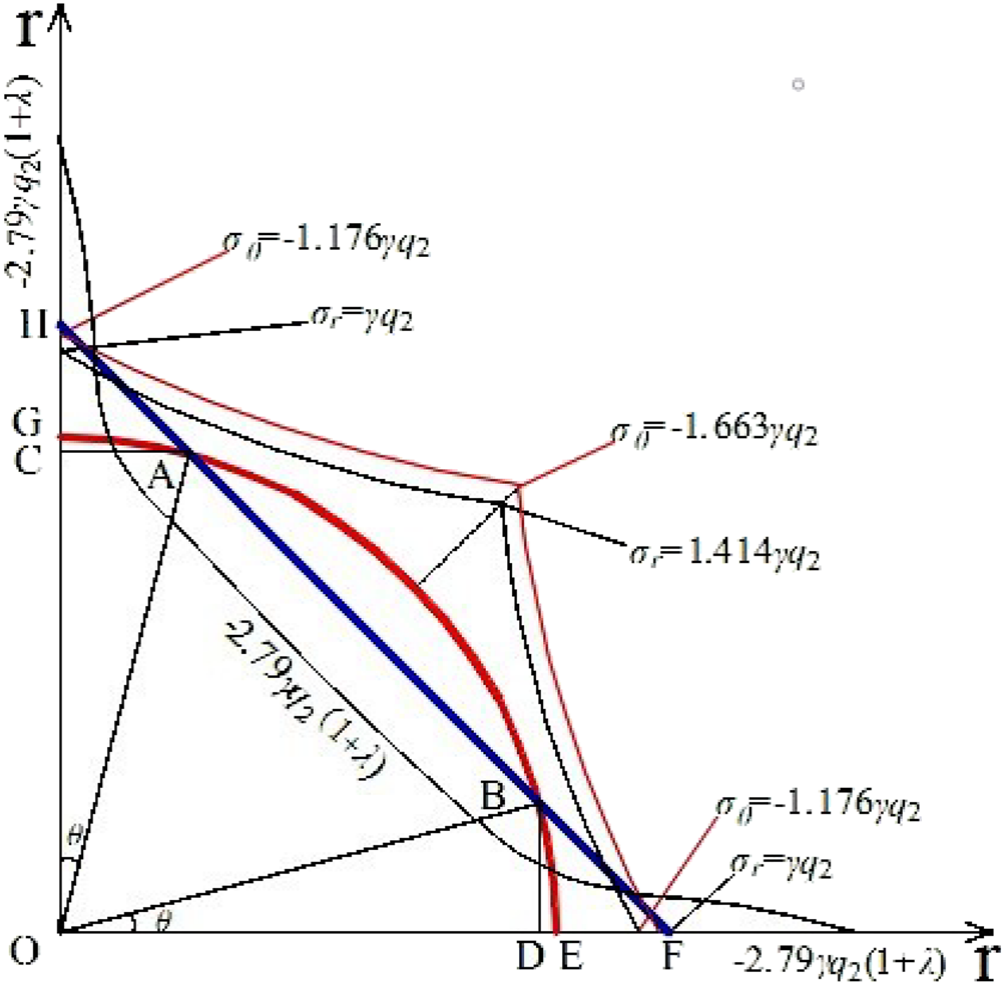
Distribution of *σ*_*θ*_ around the empty hole wall.

The coordinate system was established as shown in Fig. 4, and the data were brought into Eqs. (1) and (4) to make the stress plot.

### Equivalent structural mechanics model

**Figure 5:**
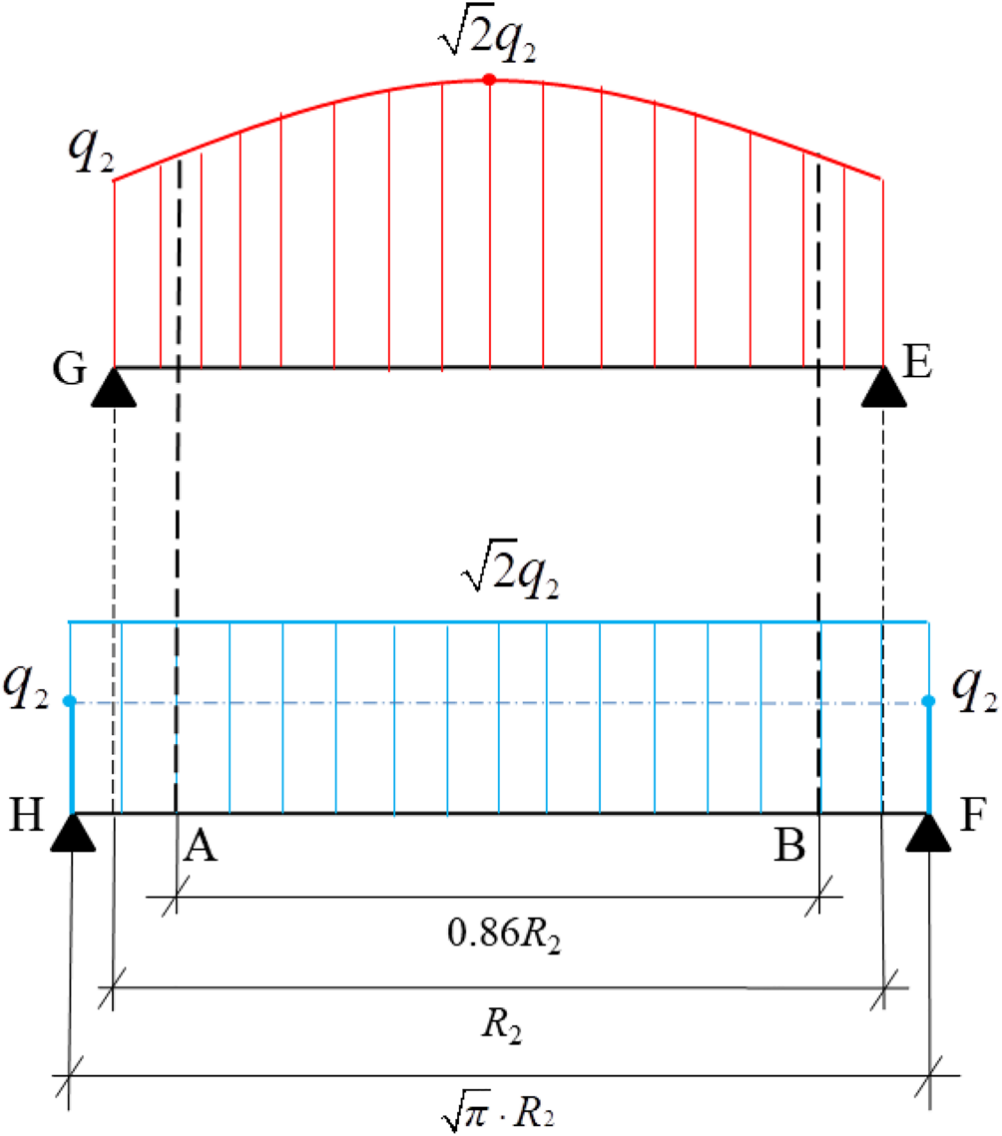
Load distributions of simply supported beams with equivalent rigid joints.

Let the radius of the circular empty hole be 1, then, *a*: *R*_2_ = π^0.5^. The calculated coordinate points in Fig. 4 are A (0.299, 0.955), B (0.955, 0.299), C (0, 0.955), D (0.955, 0), E (1, 0), F (1.253, 0), AB = 1.351, BF = 0.211, and *a* = 1.772. The rigid body structure shown in Fig. 5 is established.

From the length of the action line, in the same cross-sectional area of the circle empty hole and square empty hole, the square empty hole is better than the circular empty hole. When the stress wave on the empty hole wall creates the Hopkinson effect, the stress on the wall of the square empty hole transmits so in the same broken area, which will cause the rock mass to form a larger fracture.

As shown in Fig. 5, since the strength of the stress wave is much greater than the tensile and shear strengths of the rock mass, on section GE, according to formula (1), the radial stress and annular stress of the circular holes wall are basically the same, and the stress change rate for* θ* = (0, 0.5π) is sin *θ*. When* θ* = 0.25 π, the stress of the circular holes wall is largest, but the circular radial stress dispersed to the ring moves upward, the actual ring stress is greater than the theoretical ring stress calculation value, and the rock pressure on the wall of the circular holes decreases. According to formula (4), the calculated circumferential stress and radial stress of the square empty hole wall are smaller than those of the circular holes wall, but under the action of the square empty hole wall explosion stress wave, the stress at the corner (The HG', E'F section of Fig. 5) is concentrated, and its value is much greater than that of the GE section. Therefore, at G and E of the rock mass, shear failure occurs, while the radial and annular stresses of the rock mass in the GE section are uniform. However, because the tensile strength of the rock mass is much lower than both the shear and compressive strengths, and the tensile stress of the HG 'and E’F sections are much greater than that of G'E'.

### Circular empty hole wall mechanical characteristics

The hoop stress *σ*_*θ*_ squeezes the surrounding rock at the holes wall^22^. When the circular wall is subjected to circumferential stress, the radial stress *σ*_*r*_ is dispersed on the circular wall, and the additional circumferential stress generated will prevent the radial shear stress *τ*_*θr*_ from cutting the rock mass and resist damage to the circular wall^23^.

**Figure 6:**
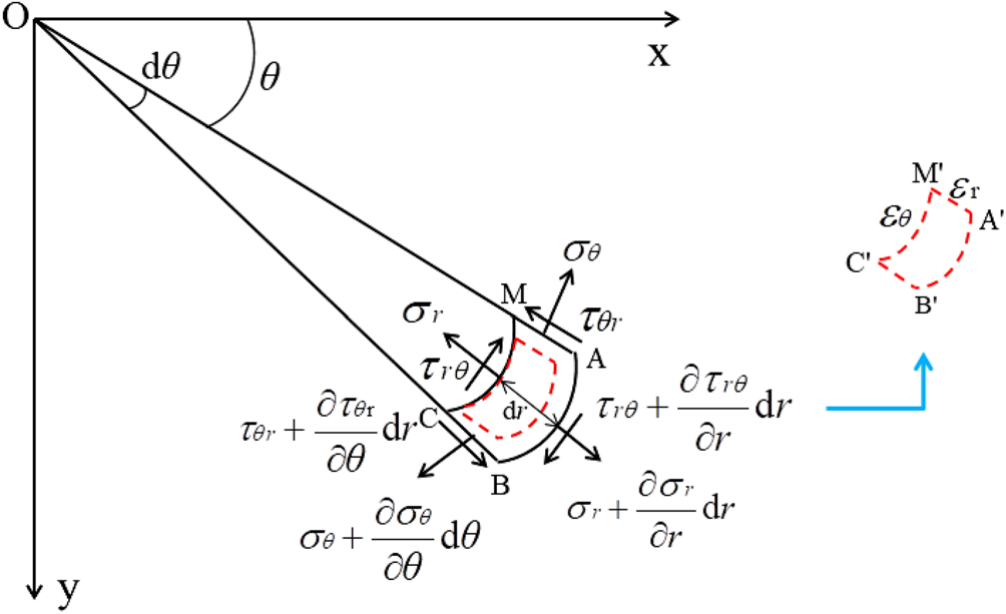
Planar polar coordinate system of microelement M with a circular void.

To establish the circular empty hole wall microelement body as shown in Fig. 6. *σ*_*r*_ is the radial stress, *σ*_*θ*_ is the hoop stress, and *τ*_*rθ*_ and *τ*_*θr*_ are shearing stresses. Since the stress varies with the coordinate *r*, increments were generated in the micro body: *σ*_*r*_ + (∂*σ*_*r*_ / ∂r) × d*r*, *σ*_*θ*_ + (∂*σ*_*θ*_ / ∂θ) × d*θ*, and *τ*_*rθ*_ + (∂*τ*_*rθ*_ / ∂r) × d*r*, where positive stress is compressive (radial and hoop) or clockwise (shear) in the coordinate system. The radial thickness of the microelement is d*r*, and the ring angle is d*θ*. When the mass point is stressed, it generates strain from the planar ABCM to A'B'C'M'.

Considering the radial and cyclic displacement of the microelement, assume *u*_*r*_ = *MM*',* u*_*r*_ + (∂*u*_*r*_/∂*r*) × d*r* = *AA*', *u*_*r*_ + (∂*u*_*r*_/∂*θ*) × d*θ* = *BB*', *u*_*θ*_ = *MM*', *u*_*θ*_ + (∂*u*_*θ*_/∂*r*) × d*r* = *AB* − *AB*', and *u*_*θ*_ + (∂*u*_*θ*_/∂*θ*) × d*θ* = *CC*'. Obtain the following physical equations:

5 $$\left\{ \begin{gathered} \varepsilon r = \frac{{1 - \nu^{2} }}{E}\left( {\sigma r - \frac{\nu }{1 - \nu }\sigma \theta } \right) \hfill \\ \varepsilon \theta = \frac{{1 - \nu^{2} }}{E}\left( {\sigma \theta - \frac{\nu }{1 - \nu }\sigma r} \right) \hfill \\ \gamma r\theta = \frac{2(1 + \nu )}{E}\tau r\theta \hfill \\ \end{gathered} \right.$$

Let the critical elastic strain before the crushing of the rock mass be [*ε*_*r*_] and [*ε*_*θ*_] and the critical stress be [*σ*_*θ*_]. Making *u* = (1 + *ν*)/*E*, Eq. (5) is improved to derive the expression for [*σ*_*θ*_] and [*ε*_*r*_]:

6 $$[\sigma \theta ] = \frac{{1 - \nu_{{\text{d}}} }}{{\nu_{{\text{d}}} }}[\varepsilon \theta ] + \frac{{(2u - 2)\nu_{{\text{d}}}^{2} + (2 - 3u)\nu_{{\text{d}}} + \nu_{{\text{d}}} - 1}}{{ - 2u\nu_{{\text{d}}}^{2} + u\nu_{{\text{d}}} }}[\varepsilon r]$$

where [*ε*_*θ*_] > *ε*_*θ*_ > 0, [*ε*_*r*_] > *ε*_*r*_ > 0, When the rock mass is compacted, the lateral shrinkage coefficient ν decreases. According to relation (13), the coefficients of [*ε*_*r*_] and [*ε*_*θ*_] in the expression [*σ*_*θ*_] increase as ν decreases.

## Numeral simulation and analysis

### Model building

**Figure 7:**
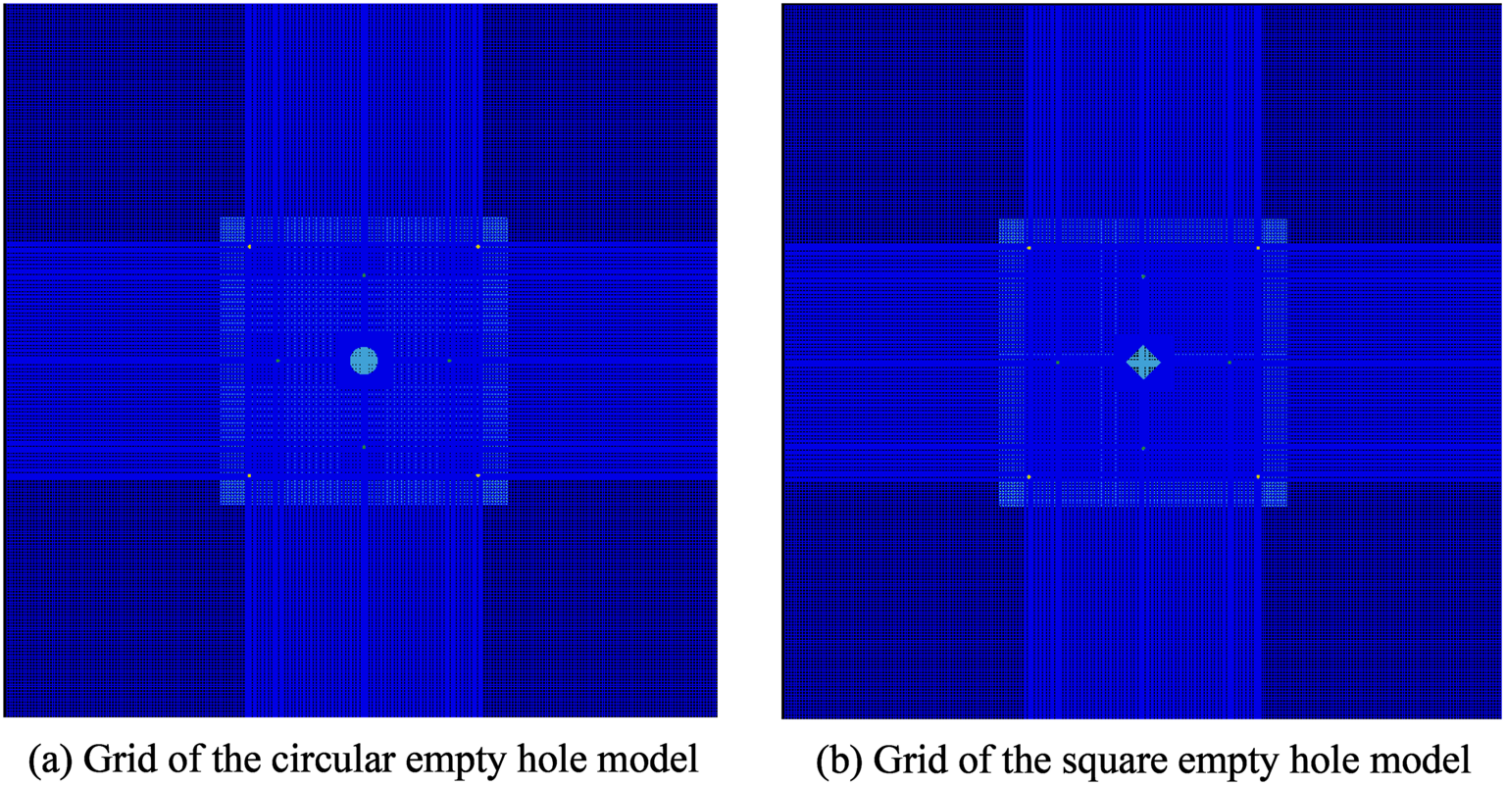
Numerical model of a cut holes with an empty hole.

Through the numerical simulation software ANSYS/LS-DYNA building a two-dimensional model with a size of 5 m × 5 m (side length), the algorithm of flow-solid coupling of Multi-Substance ALE is used to solve the problem of nonlinear dynamics of large deformation. As shown in Fig. 7, the circular empty hole model is divided into 164,767 units, and the square empty hole model is divided into 145,267 units. The side length of the cut area is 1.65 m, 1.675 m from the model boundary, and the model boundary around no reflection^24,25^.

The rock model adopts the *MAT_PLASTIC_KINEMATIC keyword in LS-DYNA, and the parameters are shown in Table 2.

**Table 2:** Parameters of the rock model.

Density	Poisson's ratio	Compressive strength	Tensile strength	Shear strength	Modulus of elasticity
/(g∙cm^-3^)		/MPa	/MPa	/MPa	/GPa
2.74	0.35	95.7	6	13.3	25.8

Using the # 2 rock emulsion explosive as the material, the keyword *MAT_HIGH_EXPLOSIVE_BURN in LS-DYNA is used to build the models, and the parameters are shown in Table 3^26^. The blast holes diameter is 32 mm using coupling loading. In the process of explosion, the detonation pressure and the relative volume of the detonation product are described using the JWL equation of state:

7 $$P = A\left( {1 - \frac{\omega }{{VR_{1} }}} \right)e^{{ - R_{1} V}} + B\left( {1 - \frac{\omega }{{VR_{2} }}} \right)e^{{ - R_{2} V}} + \frac{{\omega E_{0} }}{V}$$

**Table 3:** Exploded material parameters.

Density	Detonation velocity	Detonation pressure	*A*	*B*	*R*_1_	*R*_2_	*ω*	*E*_0_
/(g∙cm^-3^)	**/**(m∙s^-1^**)**	/GPa	/GPa	/GPa				/GPa
1	4500	27	276	0.84	5.20	2.01	0.50	3.87

*P* is the initial detonation pressure during the detonation process in MPa; *V* is the explosion product volume; and *L* and *E*_0_ are the initial specific internal energies of the detonation product. *A*, *B*, *R*_1_, *R*_2_, *ω* and *E*_0_ are the parameters related to blasting, and the basic parameters of the explosives are shown in Table 3.

### Model reliability validation

The numerical simulation is mostly compared with the results of Zhang et al.^4^. The reliability of the model was verified by single holes blasting experiment. Through five single-holes blasting tests, the data of the number of main cracks, the length of crack propagation and the width of the lamellar crack zone were obtained. PMMA was used as an alternative material, which showed good brittle failure characteristics under explosion. The design size of the glass plate is 500 mm × 500 mm × 5 mm. The data are presented in Table 4.

**Table 4:** Single hole blasting test.

	Test 1	Test 2	Test 3	Test 4	Test 5	Mean value
*n*	8.286	7.990	8.615	8.823	8.516	8.445
*ω*	0.446	0.425	0.414	0.463	0.409	0.432
*z*	0.142	0.126	0.133	0.118	0.122	0.128

**Figure 8:**
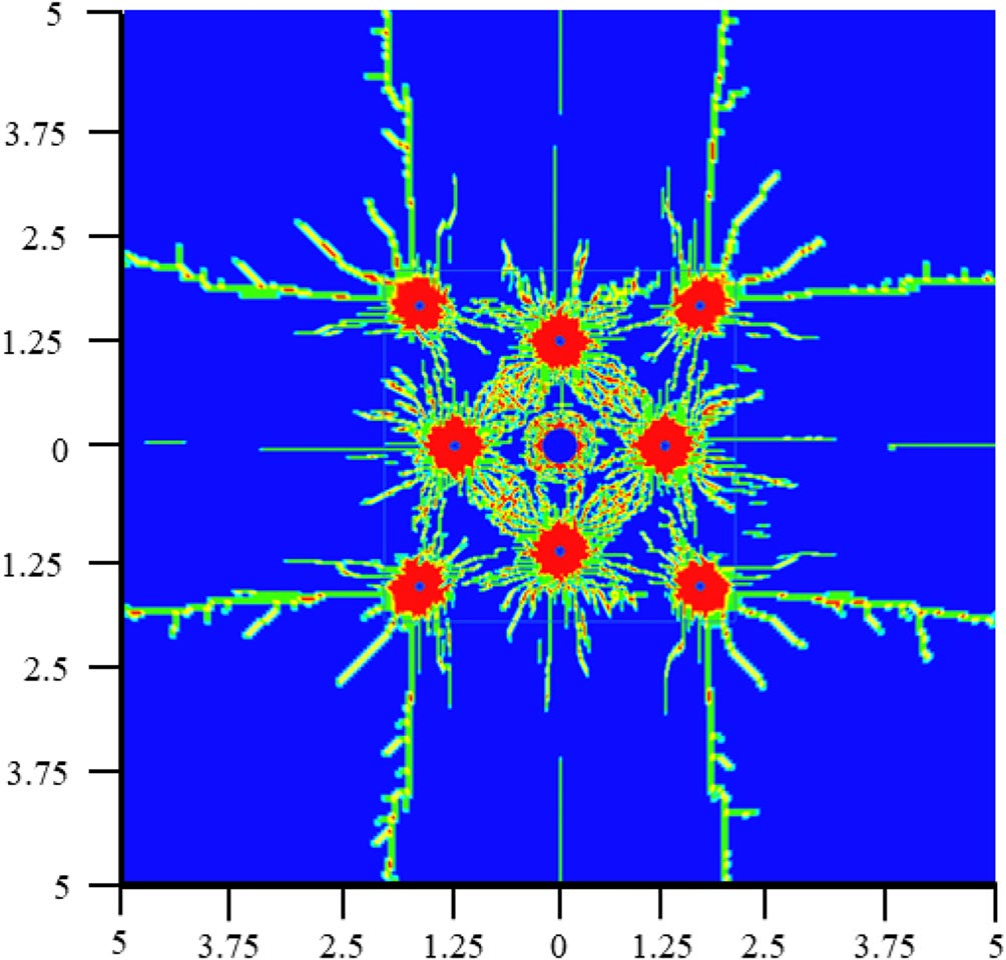
Length of damage crack in circular open holes.

The average of the main crack was *n* = 8.445, the average crack growth length was *ω* = 0.432 m, and the average crack width was *z* = 0.128. In the numerical model (Fig. 8), the mean value of the main crack is *n* = 8.421, the crack propagation length is *ω* = 0.426 m, and the width of the laminar crack region is* z* = 0.134.

**Figure 9:**
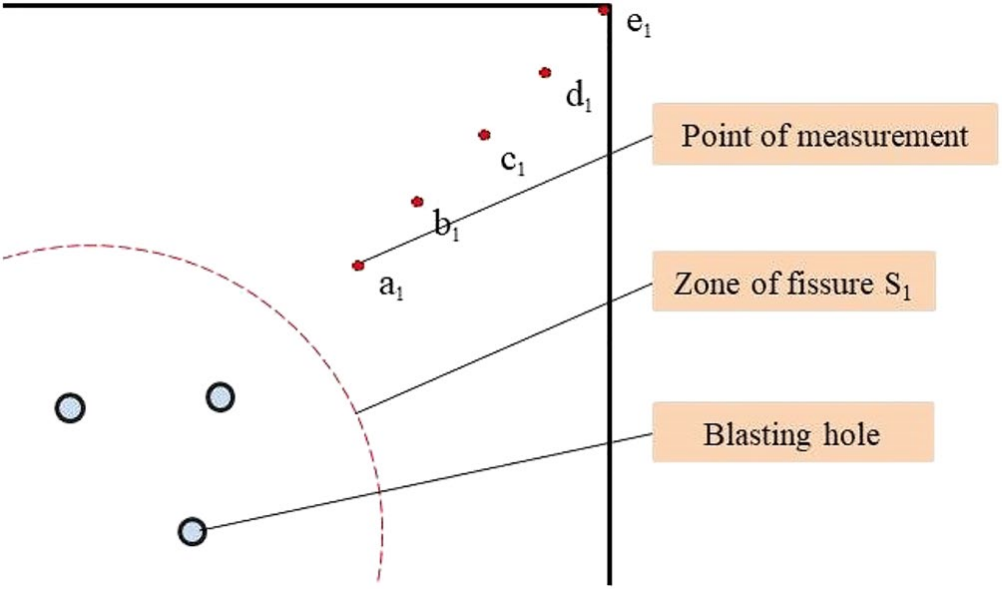
Rock mass vibration velocity monitoring point in elastic zone.

**Figure 10:**
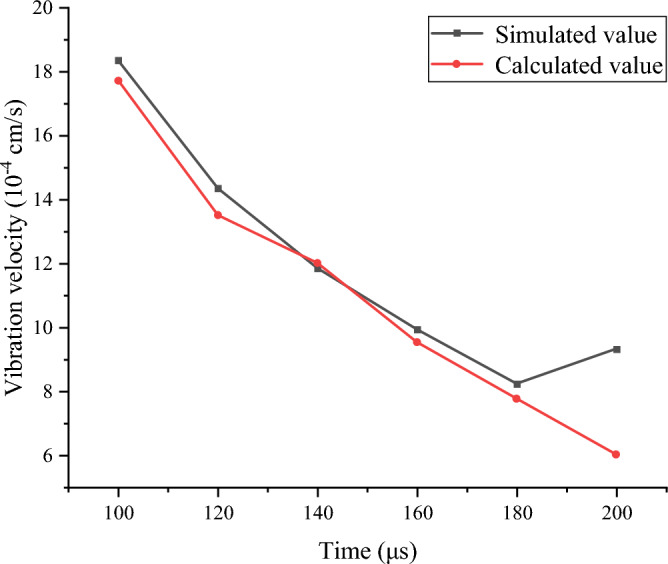
Vibration velocities of simulation and theoretical calculation.

As shown in Figs. 9 and 10, six points are selected evenly between the empty hole and the blast holes: A, B, C, D, E and F, and the law of vibration speed decay are compared. It can be seen from the figure that the simulated values of this model are consistent with the trend of changing values calculated through the Sadoski formula. The increase in the simulated value at 200 μs is because the F measurement point rock mass on the empty hole wall is on the free surface and is affected by the reflected stress waves.

Through the verification of the above two aspects, the numerical model has a certain reliability.

### Empty hole mass point displacement

**Figure 11:**
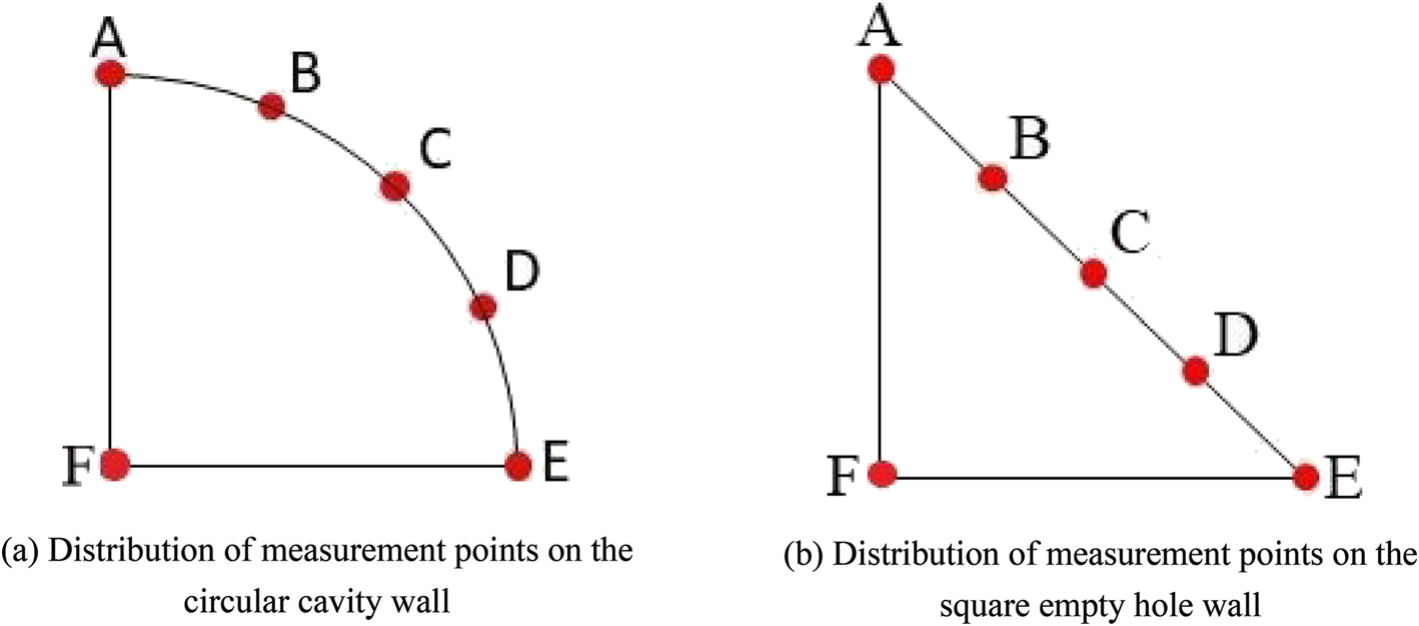
Measurement point of empty hole wall displacement.

To monitor the displacement of the empty hole, the measurement points as shown in Fig. 11. A, B, C, D, and E are the empty hole wall monitoring points, and F is the empty hole central vibration monitoring point.

**Figure 12:**
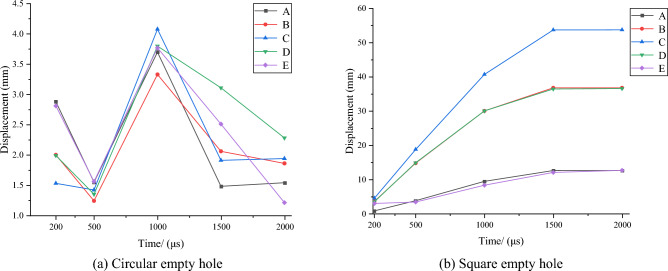
Displacement of the empty hole wall.

The displacement during the explosion is shown in Fig. 12. On the wholes, the displacement of the square empty hole wall at B, C and D is much greater than that of the circular empty hole wall. The maximum displacement point C of the circular empty hole wall is 4.08 mm, and the maximum displacement point C of the square empty hole wall is 53.74 mm. Due to the stress concentration at the corner of the square empty hole wall, the stress on the nearby rock mass is not enough to destroy it. Therefore, the displacement of the empty hole wall measurement points A and E is less than 0.85 mm.

**Figure 13:**
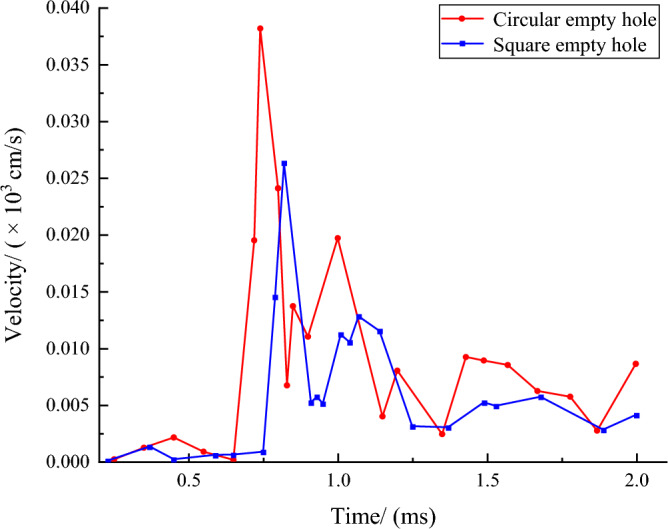
The vibration velocity of the empty hole center region changes with time.

In order to explore the energy loss of the explosion in the empty hole region, the vibration of the empty hole region was monitored. Among them, the vibration energy dissipation of circular and square empty hole to the center of the cutting area is shown in Fig. 13.

Figure 13 shows that under the action of the first and second stress waves, the maximum vibration velocity of the circular empty hole is 38.2 cm/s and 19.7 cm/s, respectively. The maximum vibration velocity of the square empty hole was delayed by 80 μs than that of the circular empty hole, which was 26.3 cm/s and 12.8 cm/s. Respectively, the energy utilization ratio of the circular empty hole cut model is lower than that of the square empty hole. After *t* = 1,000 μs, the vibration of the circular empty hole region showed an obvious oscillation pattern, and the average residual vibration was 0.824 cm/s higher than that of the square empty hole.

### Stress distribution trend

As an important indicator of rock mass damage, the equivalent stress plays a vital role in the destruction of the rock mass. In the model, the stress distribution of the internal rock mass under the action of a stress wave is represented in the form of a stress contour^27^. As the deformation index of rock material, the minimum partial stress also plays an important role in rock mass damage^7,24,25,28^.

**Figure 14:**
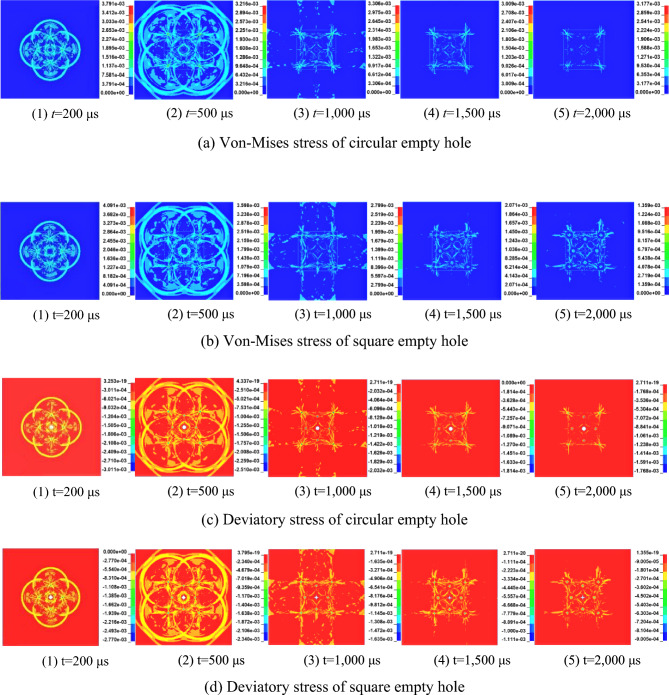
Changes in equivalent stress and minimum deviatory stress. Note: Stress in the model: 10^5^ MPa.

Figure 14 shows the contour diagrams of the equivalent stress and minimum partial stress of the circular empty hole model and square empty hole model rock masses under the action of a stress wave.

The explosion time was 200 μs, 500 μs, 1,000 μs, 1,500 μs and 2,000 μs. The contour distributions of the two contour maps are consistent, indicating that the rock mass away from the fracture zone under the stress wave appears as a tensile–shear crack^29^, resulting in substantial damage and deformation of the rock mass. The following can be concluded from the stress contour maps of the two models.

S_2_ area: The rock mass stress distribution of the square empty hole model is uniform, and the stress distribution between the blast holes and the empty hole is continuous. However, the stress distribution of the circular empty hole model rock mass is intermittent, with a local uneven phenomenon, and the stress is discontinuous.

Outside the groove area: After 1,000 μs, under the action of the second stress wave, the stress distribution of the square empty hole model is more concentrated than that of the circular empty hole model, the action time is longer, and the damage site is larger and more severely damaged.

**Figure 15:**
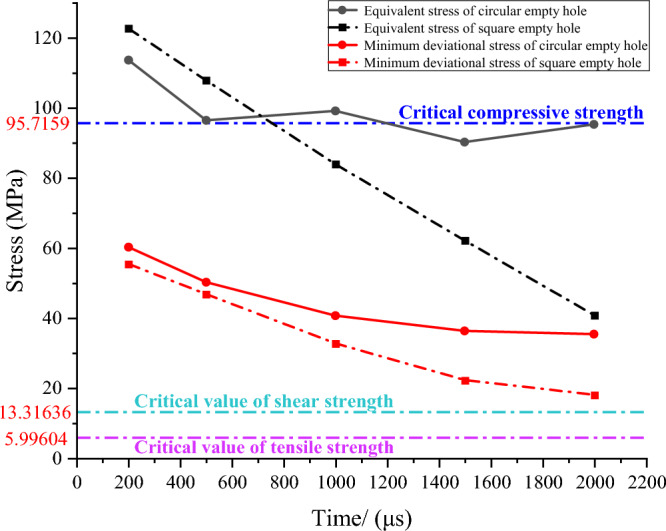
Time history curve of the mean stress change.

The explosion shock wave in the blast holes crack zone will crush and crack the rock mass, but the rock outside the elastic zone is broken by attenuated stress waves. The average stress-time history curve of the stress as shown in Fig. 15.

It can be seen from the figure that the deviatory stress of the rock mass away from the fissure area in the model is greater than the shear strength and tensile strength of the rock mass. After 500 μs, the equivalent stress magnitude is basically less than the critical compressive strength of the rock mass, the destruction of the rock mass is affected by the deviatory stress, the square empty hole model stress changes for a long time, and the tensile-shear range is large.

### Damage and crack analyses of rock blasting holes with a central empty hole

When an explosion occurs, the rock mass within 3 to 5 times the aperture around the blast holes is crushed by the shock wave, and the rock mass within 10 to 15 times the aperture produces radial cracks^30,31^. Afterward, with the decay of the explosion energy, the shock wave is converted into a stress wave and propagates in the rock mass. At this time, the influence of the stress wave is not enough to crush the rock mass^31,32^. The shear strength is higher than the tensile strength of the rock mass, resulting in tensile damage and continuous expansion of the rock mass^33^.

**Figure 16:**
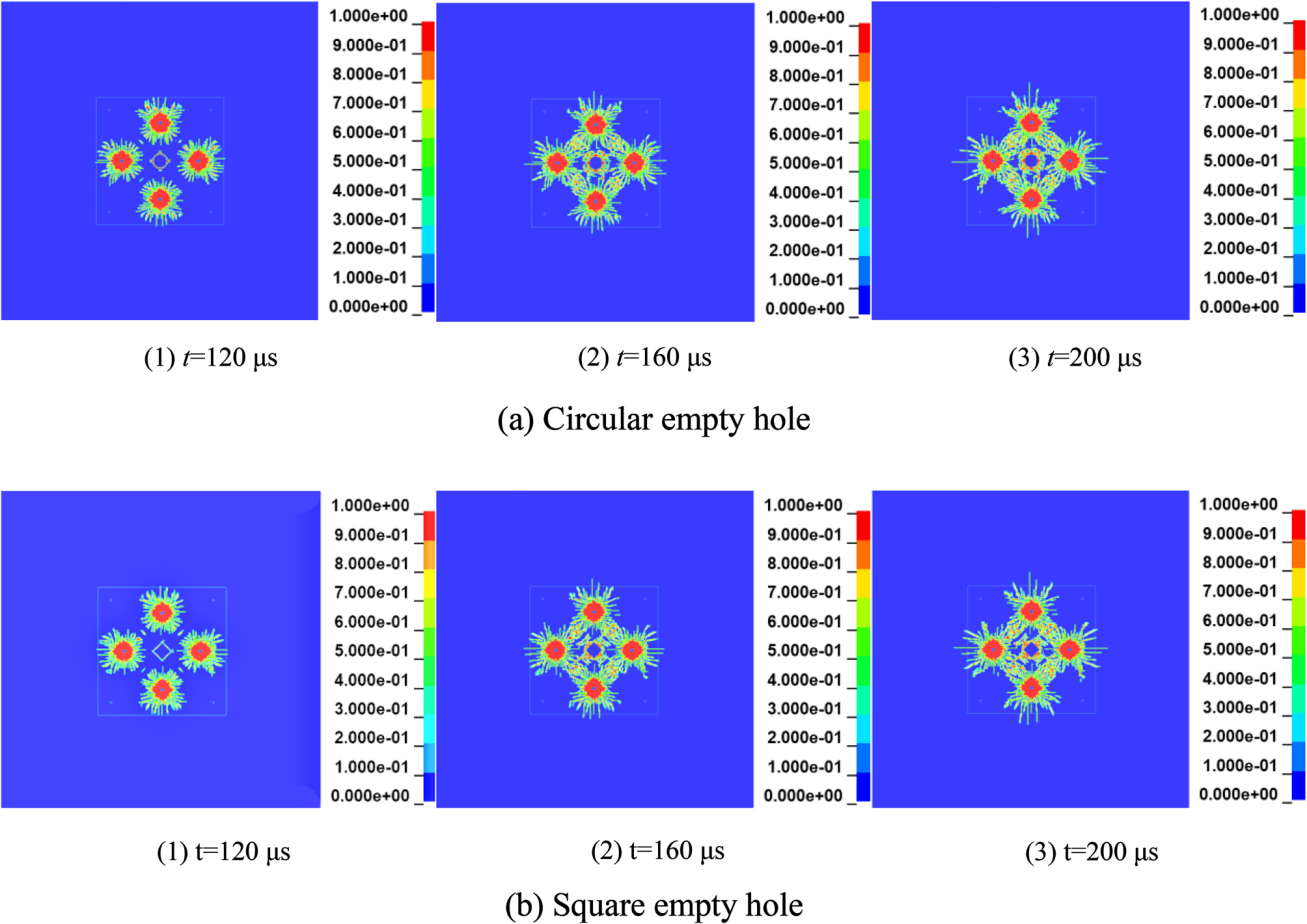
The first stage of empty hole cutting damage history.

Figure 16 shows the initial damage process of the empty holes wall in the first stage blasting holes. When *t* = 120 μs, the circular empty hole wall is initially damaged by the stress wave, the strains in the horizontal and vertical directions are the largest, and the rock mass force of the vertical holes wall is uniform. It can be seen from the damage diagram that the rock mass damage in a certain range near the empty hole wall is annular, indicating that the empty hole wall conforms to the cylinder theory under the action of stress waves. When *t* = 160 μs, the reflected stretching wave causes the "interval layer" crack in the vertical direction of the square empty hole wall. With the development of layered cracks, under the collision of stress waves in the rock mass, the interval layer cracks gradually connect with the cracks produced by the blast holes, the empty holes wall collapses inward, and the range of the empty holes increases.

**Figure 17:**
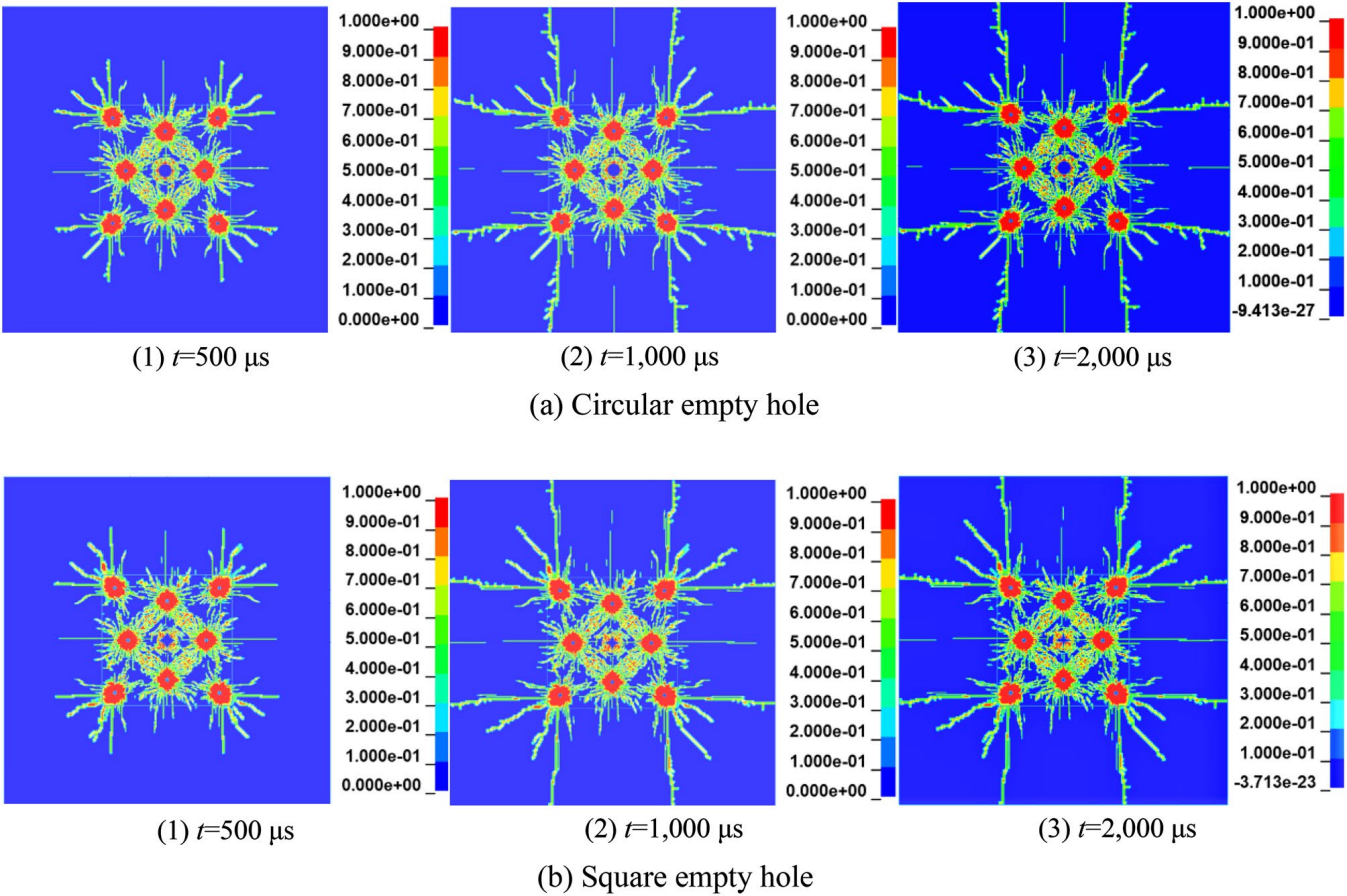
Damage history of the rock mass in the second stage of blasting.

The damage effect of the second-stage stress wave on the rock mass is based on the one-stage stress wave, and the damage results of the circular empty hole and square empty hole are as shown in Fig. 17. When *t* = 500 μs, under the action of the second stage stress wave, due to the small shrinkage of the empty hole under the action of the first stage stress wave. When the second stage stress wave acts on the original damage, the circular empty hole wall collapses slowly under the action of the reflected tensile wave. The stress of the rock mass is constantly redistributed over time. When *t* = 500—1,000 μs, the second stage stress wave makes the rock mass produce cracks in the corner of the square empty hole S_2_ area. When* t* = 1,300 μs, the square empty hole model continuously exhibit more damage, the damage development of the circular empty hole model is completed at this time, and the square empty hole model vertical crack length is 1.4 times that of the circular empty hole model.

In summary, due to the change in the shape of the empty hole, the stress concentration in different areas of the rock mass changes and the impedance of the square empty hole wall is less than that of the circular empty hole wall. The empty hole undergoes rapid inward collapse, and the evolution of the stress distribution leads to the failure of the groove area of the rock, which can also cause more damage to the rock mass, even in the case of a low stress concentration.

### Results and analysis

The algorithm structure of the computational and predictive models in this study is shown in Fig. 17.

**Figure 18:**
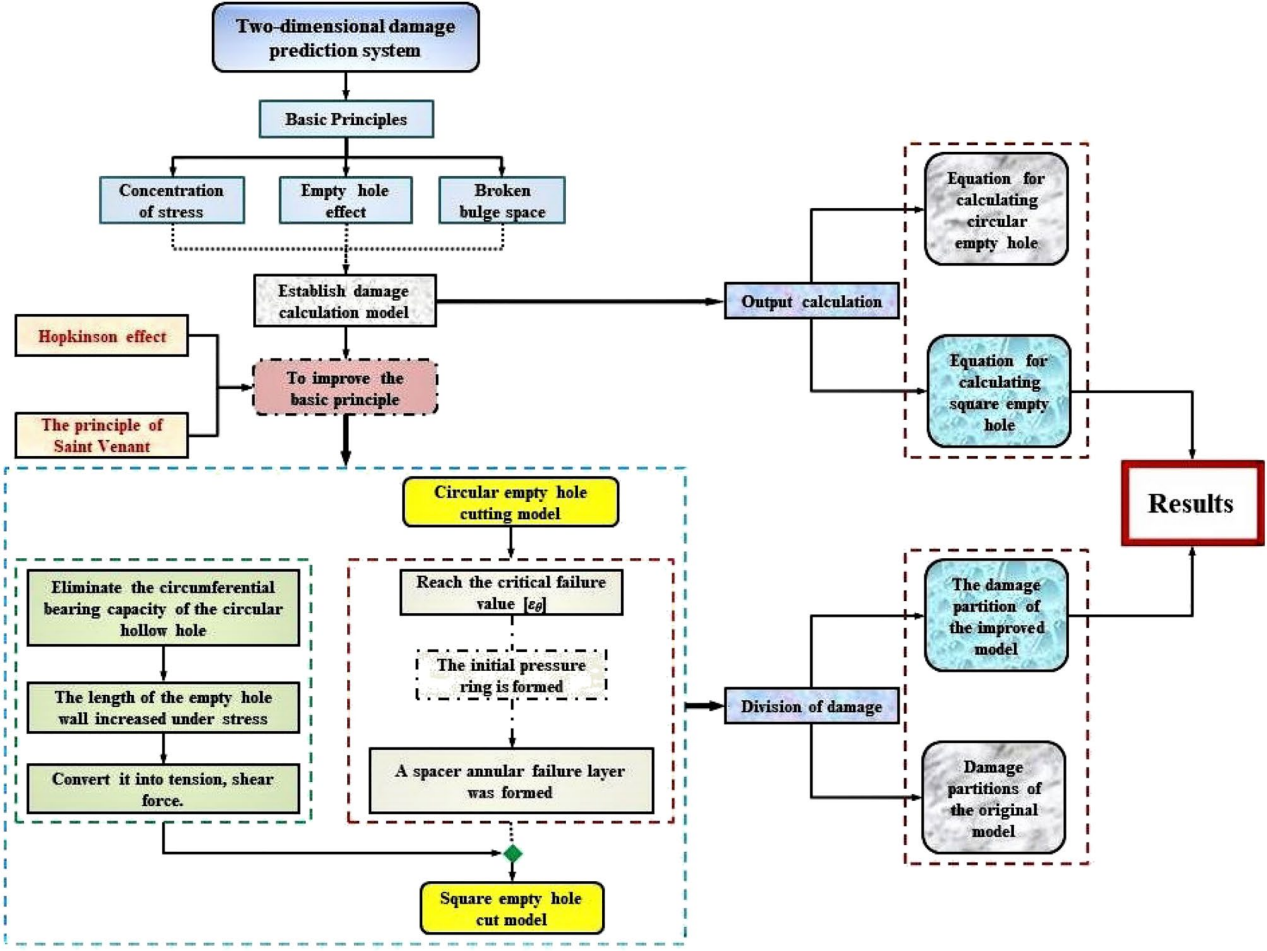
Cut blasting damage algorithm structure.

According to the calculation, prediction and numerical simulation of the theoretical model, the tension and compression partitions of the cutting area of circular empty hole and square empty hole are established by the algorithm structure, as shown in Fig. 18, 19. This is basically the same trend of stress distribution as the simulation results in Figs. 13, 15, and 16).

**Figure 19:**
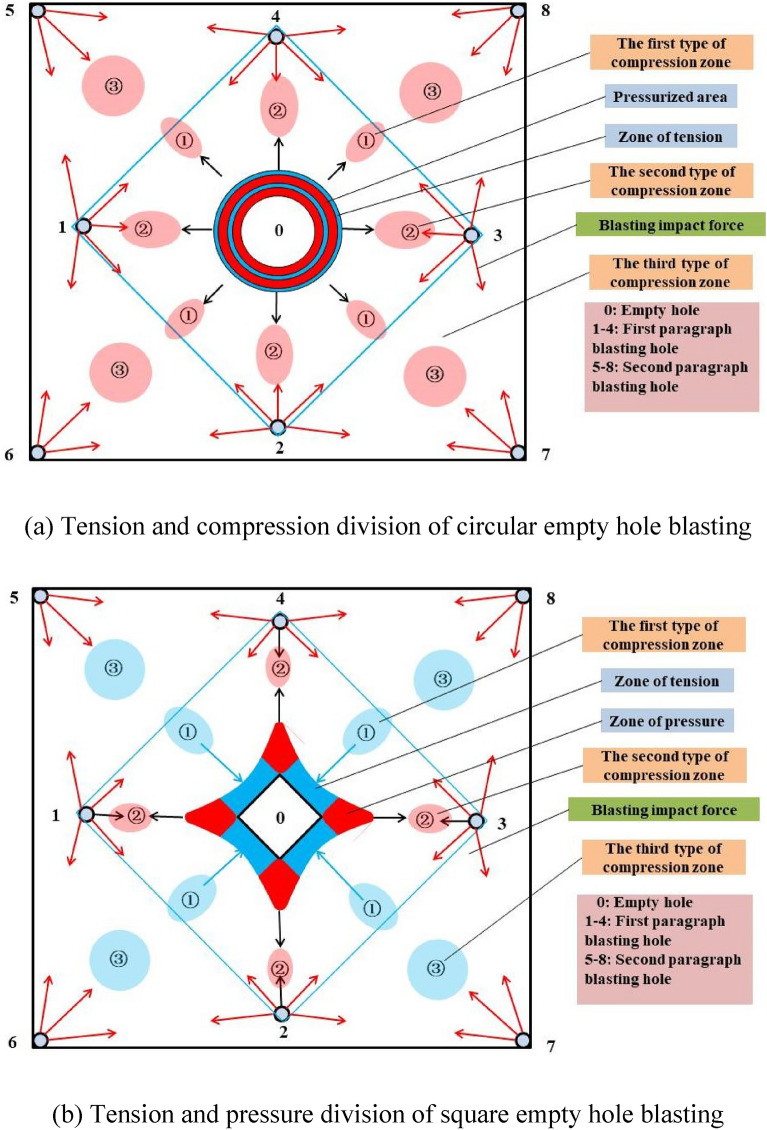
Distribution of the tensile and compressive damage of the rock mass.

Areas 1, 2, 3 and 4 are enclosed by the blast holes connection in the circular empty hole, the square empty hole model shows fragmentation of the rock mass at A, and area B is set around area A. The analysis is as follows:

Rock mass damage in area A: (a) In area ①, where the impedance of the crack development is inhibited between the circular empty and blast holes. (b) in area ①, where the collapse of the square empty hole is conducive to the continued development of cracks between the blast holes. (a) in area ②, where the impedance of the empty hole inhibits the crack expansion of areas 1, 2, 3 and 4 to the empty hole. (b) in area ②, where the stress concentration in the corner of the square empty hole develops the expansion of cracks in the rock mass at the connection between the blast holes and the square empty hole in areas 1, 2, 3 and 4. According to the displacement data of the mass point of the empty hole wall, the displacement of the first square empty hole is basically the same as that of the connection, but the damage effect of the square empty hole is better than that of the circular holes.

Rock mass damage in area B: Based on the blasting in area A, (a) In area ③, the rock mass is highly resistant to areas ① and ②, and the crack expansion is inhibited. (b) in area ③, on the basis of the tensile and shear failure of the rock mass in areas ① and ②, the square empty hole rapidly collapses inward, and the longitudinal and transverse cracks of the rock mass between the blast holes and the empty hole continue to expand, so that the stress of the square empty hole model after 1,000 μs is larger than that of the circular empty hole model for a long time.

In general, the square empty hole model works better than the circular empty hole model in terms of damage distribution range and time of action. The crack extension trend of the circular empty hole model agrees with that from the studies by Yang et al.^13^ and Li et al.^6^.

## Conclusions

1. When the rock mass with circular empty hole wall is subjected to stress, it produces resistance and is not conducive to failure. In the relationship between the circumferential strain, radial strain and circumferential stress, radial stress in the critical failure state, it is found that the radial stress contributes to the circumferential stress. The length of force on the wall of a square empty hole is 1.77 times larger than that of a round hole under the same bulging space. With the evolution of dynamic stress, the rock mass near the circular empty hole wall appears "layered annular failure" in a certain range, while the rock mass around the square empty hole wall shows multiple layers tensile damage, and the damage lasts for a long time. In terms of empty hole space, the amount of wall collapse of the square empty hole is 8.5 times that of the circular empty hole cut model.
2. Due to the circumferential impedance of the circular empty hole, the work of the explosion stress wave on the rock is consumed on the multi-layer extrusion and crushing of the circular empty hole wall. However, the explosion stress wave of the square empty hole cut holes is mostly used as the stretching work of the rock mass. The results are as follows: the ratio of wasted work of the circular holes cutting blasting model is higher than that of the empty hole model. Moreover, the residual vibration generated by the circular empty hole model is about 0.824 cm/s higher than that of the square empty hole model, which is not conducive to the shock reduction and protection of the blasting.

This study analyzes a model based on the abovementioned constraints, ignoring the stress wave at the holes wall in the first 10 μs, finding that the holes wall stress calculation accuracy has certain limitations. It only shows the transfer of the holes wall stress to the surrounding rock mass. In the future, we can further quantify the analytical process of holes wall stress change with time.

## Data Availability

The data that support the findings of this study are available from the corresponding author upon reasonable request.
